# AI-Integrated Micro/Nanorobots for Biomedical Applications: Recent Advances in Design, Fabrication, and Functions

**DOI:** 10.3390/bios15120793

**Published:** 2025-12-02

**Authors:** Prashant Kishor Sharma, Chia-Yuan Chen

**Affiliations:** Department of Mechanical Engineering, National Cheng Kung University, Tainan 701, Taiwan; n18127038@gs.ncku.edu.tw

**Keywords:** artificial intelligence in biosensing, microfabrication, microfluidics, microrobots, nanostructured biosensors, organ-on-chip, point-of-care diagnostics

## Abstract

The integration of artificial intelligence (AI) and micro/nanorobotics is fundamentally reshaping biosensing by enabling autonomous, adaptive, and high-resolution biological analysis. These miniaturized robotic systems fabricated using advanced techniques such as photolithography, soft lithography, nanoimprinting, 3D printing, and self-assembly can navigate complex biological environments to perform targeted sensing, diagnostics, and therapeutic delivery. AI-driven algorithms, mainly those in machine learning (ML) and deep learning (DL), act as the brains of the operation, allowing for sophisticated modeling, genuine real-time control, and complex signal interpretation. This review focuses recent advances in the design, fabrication, and functional integration of AI-enabled micro/nanorobots for biomedical sensing. Applications that demonstrate their potential range from quick point-of-care diagnostics and in vivo biosensing to next-generation organ-on-chip systems and truly personalized medicine. We also discuss key challenges in scalability, energy autonomy, data standardization, and closed-loop control. Collectively, these advancements are paving the way for intelligent, responsive, and clinically transformative biosensing systems.

## 1. Introduction

The growing complexity of biomedical challenges has sparked a new era of innovation, defined by the emergence of compact, multifunctional platforms tailored for high-resolution diagnostics, targeted therapeutics, and real-time biological monitoring [1,2,3]. While traditional analytical systems have long been employed as foundational tools in molecular biology and healthcare, their applicability has been constrained by inherent limitations, including high reagent consumption, labor-intensive workflows, and prolonged processing times [2,4]. As a result, throughput has often been compromised, particularly in point-of-care and resource-limited settings [5,6]. Moreover, the inability of conventional platforms to resolve microscale biological phenomena has limited their effectiveness in applications requiring high spatial and temporal precision [3,7].

To address these challenges, micro- and nanofabrication techniques have been harnessed to construct static biosensing devices and to enable the development of mobile, intelligent micro/nanorobots capable of navigating biological environments [8,9,10]. Fabrication strategies such as photolithography, soft lithography, 3D printing, and nanoimprinting have facilitated the creation of intricate microarchitectures, which are increasingly being employed in robotic elements for fluidic navigation, localized sensing, and active cargo delivery [11,12,13]. These systems benefit from enhanced surface-to-volume ratios, engineered microenvironments, and tailored responsiveness to chemical, thermal, or magnetic stimuli, ideally suited for high-efficiency biosensing tasks [14,15,16,17].

Building on these technological foundations, researchers have integrated biological recognition elements, such as antibodies, nucleic acids, enzymes, and living cells, into fixed platforms and autonomous microrobotic systems [10,18,19,20,21]. This has enabled the development of functional biosensing microrobots, in which specific biochemical interactions are transduced into detectable physical or chemical signals during active navigation [10,18,22,23,24,25,26]. Applications now extend to in vivo diagnostics, pathogen detection, targeted drug delivery, and cancer biomarker profiling, where micro/nanorobots offer unique advantages in spatial targeting, miniaturization, and real-time adaptability [27,28,29].

In such contexts, micro/nanostructured biosensors have demonstrated distinct advantages in specificity, adaptability, and miniaturization, thereby facilitating decentralized and real-time biological monitoring [30,31,32]. However, with the rise in complexity and resolution of these robotic systems, new challenges have emerged in data interpretation and system optimization [33,34,35]. Conventional analytical methods often fall short when applied to the dynamic, high-dimensional datasets associated with biosensing microrobots [36,37,38,39]. To address this, AI methodologies, particularly ML and DL, are increasingly being employed for tasks such as motion tracking, biosignal decoding, feature extraction, and predictive modeling of biological interactions [40,41,42]. Neural architectures such as convolutional neural networks (CNNs) and recurrent neural networks (RNNs) are now used to enhance both control and interpretation in microrobotic sensing platforms [43,44].

This AI integration is further empowered by miniaturized electronics and edge computing advances, allowing real-time, on-device data processing critical for untethered or implantable microrobotic systems operating in situ [45,46]. These AI-augmented microrobots, capable of localized computation, decision-making, and actuation, reduce dependence on cloud infrastructure, minimize latency, and improve data security, especially in wearable and remote healthcare applications [47,48,49].

Considering these converging developments, a comprehensive overview of AI-integrated micro/nanorobotic biosensors is provided in this review, covering aspects ranging from fabrication and functionalization to signal transduction and data interpretation [31,50,51,52]. Special emphasis is placed on AI’s role in enhancing microrobot autonomy, sensing resolution, and biomedical applicability in diagnostics, personalized medicine, and point-of-need monitoring [53,54,55,56]. Synthesizing current advances and highlighting critical challenges, the transformative potential of synergistic integration between AI, micro/nanotechnology, and robotics in the evolution of next-generation biosensing systems is underscored [57,58,59,60].

## 2. Fabrication Strategies of Micro/Nanodevices

Advances in micro- and nanofabrication techniques have been instrumental in enabling high-resolution, functional devices tailored for biological applications. These fabrication methods determine the structural fidelity, functional integration, and application scope of the final devices. Here, an overview of commonly used fabrication strategies is presented, followed by a comparative discussion on their resolution, cost, material compatibility, and scalability. Collectively, these methods form the manufacturing foundation upon which AI-integrated micro/nanorobotic systems can be built.

### 2.1. Photolithography

Photolithography is a powerful method of creating small patterns on surfaces, and these patterns are highly valued in the manufacture of biomedical and electronic devices. It was the creation of tiny components that enabled technologies to operate on a small scale [61,62]. Recently, AI techniques have been studied and demonstrated to provide significant benefits by altering the approach to photolithography. Conventional photolithography relied on human-generated parameters for exposing time, alignment, and pattern transfer. Ultimately, it was realized that significant limitations to photolithography arose from environmental factors and user dependency: dependence on humans created variations based on environmental conditions and material properties. AI-based methods have been introduced to overcome the limitations in photolithography caused by environmental factors and user dependency [63,64,65,66,67]. This was achieved by eliminating human oversight and by introducing optimization based on data and real-time feedback control throughout the fabrication process [68,69]. Therefore, the precision, speed, and overall flexibility of the photolithography process were significantly enhanced.

**Figure 1:**
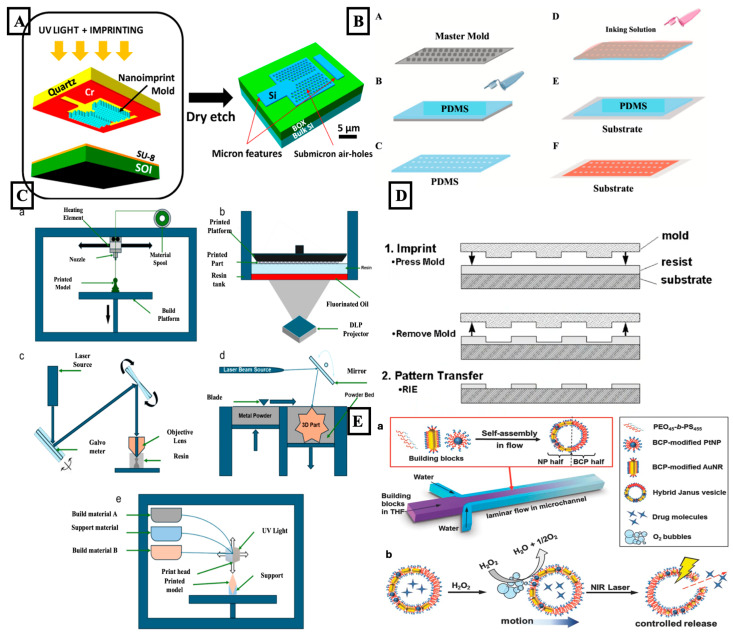
This figure presents a comprehensive overview of advanced micro- and nanofabrication strategies along with functional material assembly techniques. (**A**) Shows a schematic of the combined nanoimprint and photolithography process used to fabricate photonic crystal devices with submicron air-holes on silicon-on-insulator substrates. The figure was reproduced with permission from [71], under a Creative Commons by Non-Commercial No Derivative works (CC-BY-NC ND 4.0) license, published by MDPI, 2014. (**B**) Illustrates the steps of soft lithography-based contact printing, where subfigures A–C show the creation of a PDMS stamp from a master mold, and D–F depict inking and transfer of biomolecules to a substrate. The figure was reproduced with permission from [82], under a Creative Commons by Non-Commercial No Derivative works (CC-BY-NC ND 4.0) license, published by Elsevier, 2025. (**C**) Depicts five 3D printing methods relevant to microfluidic device fabrication: subfigure a show Fused Filament Fabrication (FFF), b illustrates Digital Light Processing (DLP), c presents Two Photon Polymerization (TPP), d details Laser Powder Bed Fusion (LPBF), and e shows Material Jetting. The figure was reproduced with permission from [98], under a Creative Commons by Non-Commercial No Derivative works (CC-BY-NC ND 4.0) license, published by Springer, 2025. (**D**) Shows the nanoimprint lithography (NIL) process, where subfigure a involves mold imprinting into a resist on a substrate, and b shows pattern transfer via reactive ion etching (RIE). The figure was reproduced with permission from [112], with a permission from WILEY-VCH, 2007, copyright license number (6075210324258). (**E**) Demonstrates the formation and function of hybrid Janus vesicles (HJVs): subfigure a describes their self-assembly from block copolymer-modified nanoparticles in a microfluidic laminar flow system, and b illustrates their catalytic propulsion in hydrogen peroxide and NIR-triggered payload release. The figure was reproduced with permission from [134], with a permission from WILEY-VCH, 2015 copyright license number (6123730217042).

AI-based computer vision algorithms enabled the automation of defect inspection and alignment correction [70]. As illustrated in Figure 1A, deep learning models trained on optical microscopy images can accurately identify line-edge roughness, resist thickness variations, and pattern distortions, outperforming traditional threshold-based methods [71,72]. Reinforcement learning was employed to optimize the exposure dose or development time, thereby minimizing process-induced deviations. These methods enabled adaptive process tuning in response to variations in substrate reflectivity, illumination uniformity, or photoresist chemistry [73,74]. Another important application of AI in photolithography comes with predictive modeling for process simulation. Neural networks trained on experimental process data predicted critical dimension outcomes under different exposure and development conditions [75,76,77]. This predictive capability reduced the need for extensive trial-and-error experiments, saving both material and time. In advanced systems, generative models produced optimized mask layouts that improved feature fidelity at sub-micron scales, enhancing reproducibility across batches.

For biomedical device fabrication, AI-enhanced photolithography contributed to higher yield and structural consistency in microelectrode arrays, biosensing platforms, and lab-on-a-chip devices. Machine learning models helped identify fabrication errors that could compromise biocompatibility or fluidic functionality [78,79]. Additionally, AI-enabled control systems monitored process drifts in real-time, enabling adaptive corrections that maintain the stability of the microstructures required for biological interfacing. However, despite these advances, various challenges still existed. The implementation of AI required significant and high-quality datasets for model training, which were often missing in academic fabrication environments. In addition, the lack of transparency within deep learning algorithms raises concerns over process traceability. Future developments are needed to integrate physics-informed AI models with empirical data and equations from the lithography process, thereby enhancing transparency and improving prediction accuracy.

### 2.2. Soft Lithography

Soft lithography has been widely utilized for fabricating micro and nanoscale features in various elastomeric materials, particularly PDMS [13,80]. While the technique offers high versatility and low-cost prototyping of biomedical and microfluidic systems, process performance is strongly dependent upon operator experience, mold quality, and curing conditions. Introduction of AI into soft-lithography workflows has minimized these dependencies by enabling automated parameter tuning, defect detection, and structural prediction [81,82,83]. The mold fidelity and replica quality were checked using AI-assisted image-processing algorithms, as shown in Figure 1B [82]. Surface defects, such as voids, channel collapse, or incomplete replication, that were previously difficult to detect by manual inspection, were identified using convolutional neural networks trained on optical and profilometric images. Machine-learning classifiers accurately differentiated between acceptable and defective microchannels, enabling improvements in fabrication yield and process consistency [84,85].

Predictive control of material processing parameters was also performed using AI models. Neural networks, trained on historical fabrication data, predicted the optimal ratios of pre-polymer to curing agent, curing temperature, and time required to achieve desired mechanical properties and dimensional stability. Reinforcement-learning frameworks dynamically adjusted these variables through the fabrication process to compensate for environmental changes, such as humidity or temperature drift. With these approaches, user-dependent variability was minimized and process optimization cycles shortened [86,87]. Furthermore, AI-driven design algorithms enabled the automatic generation of optimal microchannel geometries for specific fluidic or biological functionalities. Surrogate models using multi-physics simulations predicted the flow distribution and shear stress for organ-on-chip devices prior to actual experimental fabrication [88,89,90]. The AI-generated patterns were then transferred directly via soft lithography, ensuring that the final structures would provide results as predicted.

Even with these advances, limitations remained. AI prediction requires well-curated datasets and consistent, standardized methods for material characterization. Variability in the composition of PDMS formulations and environmental factors always presented challenges to model generalization [87,91,92]. Future work should be focused on developing transferable and physics-informed AI frameworks that can capture viscoelastic behavior, curing kinetics, and mold deformation in real fabrication environments

### 2.3. D Printing

Three-dimensional (3D) Printing, also known as additive manufacturing, is a layer-by-layer method used to create complex microscale structures from digital models [93,94]. The technique offered design flexibility and rapid prototyping, which made it valuable for microfluidic devices, biomedical implants, and lab-on-chip platforms. Among the available approaches, stereolithography (SLA), digital light processing (DLP), and two-photon polymerization (2PP) were considered as the most suitable for microscale fabrication [13,95,96]. These methods achieved element sizes from tens of micrometers down to the submicron range. However, a wide range of photocurable polymers, hydrogels, and biocompatible resins had been developed, but their use was limited by poor print kinetic efficiency, weak mechanical integrity, and the need for complex post-fabrication processing [12,97,98].

AI has been progressively integrated into 3D-printing workflows to address these limitations, and the main AI-assisted modules used in the process are summarized in Figure 1C [99,100]. Machine-learning algorithms trained on historical process data were used to estimate suitable settings for printing speed, exposure conditions, and layer thickness, thereby maintaining geometric accuracy. Reinforcement-learning methods further enabled adaptive tuning of printing parameters by responding to real-time sensor feedback, which compensated for fluctuations in nozzle pressure, resin viscosity, or illumination intensity [101,102,103]. These strategies reduced user dependency and improved consistency across printed batches. Computer-vision systems powered by AI were applied for in situ defect monitoring. Convolutional neural networks analyzed images from the build plane to detect delamination, incomplete curing, or geometric distortion. When defects were identified, printing parameters were automatically adjusted for subsequent layers, forming a feedback loop that reduced material waste and enhanced reliability [102,104,105].

AI-driven generative-design tools were also used to optimize device architectures. Deep-learning models translated functional objectives, such as structural stiffness, flexibility, or magnetic response, into printable geometries. For AI-integrated micro/nanorobotics, 3D printing offers unmatched customization potential, enabling the fabrication of device architectures optimized directly from simulation-driven AI design outputs [106,107,108]. In micro- and nanorobotics, simulation-guided AI predicted motion behavior and supported the fabrication of structures tailored for controlled navigation or targeted drug delivery. These design-to-fabrication pipelines linked virtual performance predictions with physical manufacturing, thereby improving device adaptability. However, several challenges still existed. Differences in machine configurations and print materials limited the transferability of trained models. The absence of standardized datasets restricted large-scale validation, especially at submicron resolutions achievable with 2PP. Future work was expected to focus on uniform data collection frameworks and hybrid AI–physics models that enhance interpretability and extend intelligent control across diverse printing platforms.

### 2.4. Nanoimprint Lithography (NIL)

Nanoimprint lithography (NIL) is recognized as a high-resolution and cost-efficient fabrication method, in which nanoscale patterns are transferred by mechanically pressing a nanostructured mold into a thermoplastic or UV-curable resist layer [109,110]. This approach enabled the production of features as small as 10 nm and demonstrated high fidelity across various materials, including polymers, metals, and flexible substrates. Due to its simplicity and low equipment requirements, NIL was widely adopted for fabricating biosensors, photonic structures, and flexible micro- and nanodevices. However, traditional NIL faced limitations such as mold wear, alignment errors, and process variability, especially in multilayer applications [111,112,113,114,115].

AI was increasingly incorporated into NIL processes to improve pattern fidelity and process robustness, and the main AI-assisted steps were summarized in Figure 1D. Machine-learning models trained on imprinting data were used to predict residual-layer thickness and feature deformation under different imprinting conditions [116,117,118]. Reinforcement-learning and model-predictive-control strategies were applied to adjust temperature, pressure, and imprint duration in real time, which reduced mold stress and maintained uniform replication across substrates. AI-based image-analysis tools were used to interpret interferometry and electron microscopy data. These models automatically detected defects such as incomplete filling, trapped air pockets, edge collapse, and surface contamination. Automated feedback enabled corrective actions during or after imprinting, resulting in improvements in yield and consistency [119,120,121]. AI was also used to optimize mold design. Generative models produced nanoscale mold geometries that strike a balance between structural integrity and optical or electrical functionality. When combined with finite-element and Multiphysics simulations, these designs reduced deformation during repeated imprint cycles. In micro- and nanorobotic applications, such AI-optimized NIL structures supported the fabrication of dense sensor arrays, photonic components, and textured surfaces required for precise control and signal coupling [122,123,124].

However, challenges persisted because reliable predictive modeling required large datasets that captured variability across tools, materials, and environmental conditions. The lack of standardized NIL datasets restricted model generalization across platforms. Future efforts were expected to focus on shared NIL databanks and hybrid physics-informed AI frameworks that improved reliability and supported scalable deployment across fabrication environments.

### 2.5. Self-Assembly

Self-assembly was a bottom-up fabrication approach in which molecules, nanoparticles, or polymers organized spontaneously into ordered structures through thermodynamic or kinetic driving forces [125,126]. This process enabled the formation of large-area nanostructures with minimal equipment and was widely used for constructing functional coatings, hybrid interfaces, and bioinspired nanomaterials. Although self-assembly offered high material compatibility and low cost, its outcomes were sensitive to environmental variations, making reproducibility and structural control challenging [127,128]. As illustrated in Figure 1E, AI was increasingly applied to enhance the predictive understanding and control of self-assembly behavior. ML algorithms trained on experimental and simulation datasets were used to forecast morphology, defect distribution, and assembly kinetics based on input parameters such as concentration, temperature, pH, and solvent polarity. These predictive models reduced the need for extensive trial-and-error experimentation and identified parameter spaces that generated the most ordered or functional structures.

RL frameworks were employed to optimize assembly pathways dynamically. By iteratively adjusting experimental conditions and receiving feedback from in situ optical or spectroscopic measurements, AI agents learned to achieve target morphologies, such as vesicles, micelles, or layered films, with improved yield and uniformity [129,130,131]. In parallel, computer vision methods analyzed real-time microscopy images to detect aggregation defects or phase separation, enabling the automatic adjustment of environmental parameters to maintain structural integrity. AI-assisted molecular and mesoscale simulations also accelerated the design of building blocks with programmable interactions. Deep-learning generative models created novel molecular nanoparticle surface chemistries that favored desired self-assembly configurations [132,133]. Such AI-guided designs were applied to fabricate responsive nanostructures for biosensing, targeted drug delivery, and microrobotic actuation, resulting in materials that exhibited tunable optical, magnetic, or catalytic properties.

Despite these advances, limitations continued in data standardization and model interpretability. Variability in experimental datasets and the complexity of non-equilibrium phenomena limited the generalization of AI models. Future work should focus on developing physics-informed AI approaches that integrate thermodynamic principles with data-driven learning to achieve more transparent and transferable control of self-assembly processes.

## 3. Functional Integration with Biological Systems

The integration of micro- and nanodevices into biological environments is required to be carefully coordinated with the complex behavior of living systems. Functional performance is influenced not only by structural precision but also by mechanical compatibility, biochemical interactions, and the ability to respond dynamically. These requirements are considered especially important for autonomous microrobots, which are operated in confined, heterogeneous, and constantly changing physiological conditions. AI is increasingly employed to address the nonlinear and multiscale nature of biological interactions. By using data-driven prediction and adaptive control, biological interfaces were transformed by AI from static components into systems capable of real-time interpretation and response. In this section, four key aspects of biological integration were described, ECM mimicry, biocompatibility, perfusion systems, and sensor/actuator coupling, and the ways in which AI enhanced reproducibility, adaptability, and functional relevance in each area.

### 3.1. ECM Mimicry

**Figure 2:**
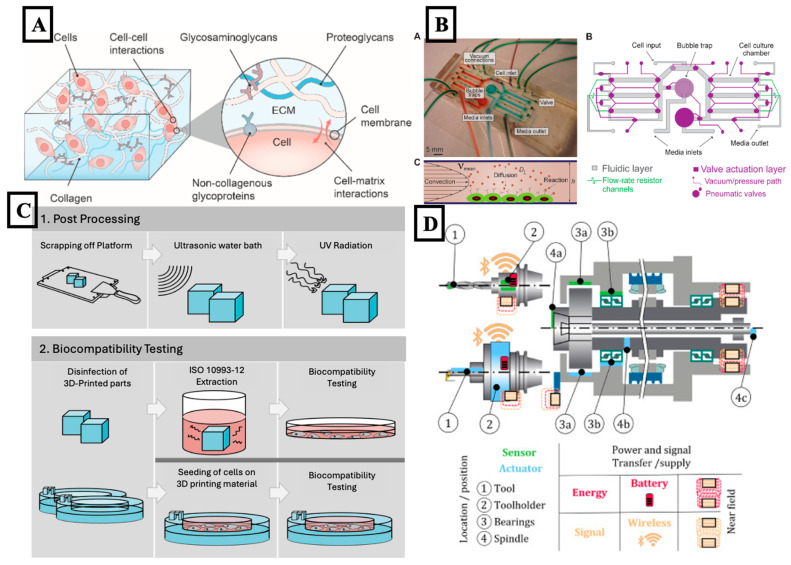
Schematic illustrations emphasizing diverse aspects of micro/nanodevice systems. (**A**) Interactions within the extracellular matrix (ECM), including cell–cell and cell–matrix communication. The figure was reproduced with permission from [136], under a Creative Commons by Non-Commercial No Derivative works (CC-BY-NC ND 4.0) license, published by Elsevier, 2024. (**B**) Microfluidic device designs and circuit-like architectures enabling controlled transport, fluid regulation, and system integration. The figure was reproduced with permission from [195], under a Creative Commons by Non-Commercial No Derivative works (CC-BY-NC ND 4.0) license, published by PLOS One, 2011. (**C**) Workflow of post-processing and biocompatibility testing of 3D-printed platforms, including sterilization, UV treatment, and cell seeding for functional validation. The figure was reproduced with permission from [196], under a Creative Commons by Non-Commercial No Derivative works (CC-BY-NC ND 4.0) license, published by IOP Publishing, 2020. (**D**) Structural and functional representation of a micro/nano-engineered platform, depicting integrated sensors, actuators, power, and signal transfer pathways for biomedical applications. The figure was reproduced with permission from [189], with a permission from Elsevier, 2023 copyright license number (6084081255416).

The structural and biochemical features of the ECM were recreated to support cell adhesion, signaling, and tissue development [135]. Engineered surface topographies, stiffness gradients, and biomimetic coatings were traditionally used to mimic ECM properties. While functional substrates were produced through these approaches, their development was largely guided by trial-and-error methods. The nonlinear relationships between surface properties and cell behavior made reproducibility difficult to achieve [136,137,138]. To address these limitations, AI was applied to predict and optimize ECM-mimetic environments. Machine-learning models were trained using imaging datasets and mechanical test results to estimate cell adhesion, spreading, and differentiation based on nanoscale topography and stiffness. Patterns that were not easily observed manually were identified, allowing improved prediction of cell behavior across various materials and geometries. Figure 2A was used to illustrate the major structural and biochemical components of the ECM that influenced cell–matrix interactions relevant to engineered microenvironments.

Reinforcement-learning methods were further used to tune scaffold fabrication parameters such as crosslinking density, fiber alignment, and porosity. Input variables were adjusted iteratively, and feedback from mechanical and biological assays was used to guide the learning process. As a result, scaffold designs were generated to better replicate native tissue mechanics. At the same time, deep-learning models were employed to analyze ECM remodeling during culture. Changes in fiber organization and matrix degradation were detected, which allowed real-time adjustment of scaffold composition or biochemical supplementation. Through these AI-assisted strategies, ECM-like platforms were made more physiologically relevant and reproducible. However, limitations remained due to variability in biological data, material inconsistencies, and limited interpretability of the model outputs. Future improvements were expected to involve the use of physics-informed AI models combined with established mechanobiological principles, enabling more transparent predictions and greater scalability in micro- and nanorobotic applications.

### 3.2. Biocompatibility

Biocompatibility was considered essential for ensuring that micro- and nanodevices worked safely within living systems. It was typically assessed using known materials, experimental testing, and long-term biological studies, both in vitro and in vivo [139,140,141]. Figure 2B is included to provide a clearer overview of the post-processing workflow used before biocompatibility assessment. These methods were time-consuming, and they often required large resources. Predicting how a material would behave over time remained difficult because of complex interactions between chemical structure, degradation, and tissue responses. To reduce this workload, AI was brought in. Neural networks were trained on existing data, including toxicity, blood compatibility, and breakdown behavior, to estimate cell-material interactions and immune reactions before building the actual device. This early prediction helped screen out materials that were unlikely to work [142,143,144].

Computer vision was also applied. Images from microscopes were analyzed to spot things like inflammation, damaged membranes, or signs of cell stress. With this setup, detection was made faster and less biased than manual observation. Optimization tools were also used, which balanced things like strength, degradation time, and how the immune system might react. These models suggested better material combinations without endless trial and error [145,146,147]. In microrobotic systems, surface coatings were evaluated using AI to reduce immune system responses during movement inside the body. Models looked at how physical forces or surface chemistry could influence macrophage recognition or trigger complement proteins. Even with these advances, there were limits. Data was inconsistent across studies. Biological tests varied, and immune systems remained unpredictable. It was suggested that better shared datasets and combined AI–mechanistic models would help improve reliability and understanding going forward.

### 3.3. Perfusion Systems (Microfluidics)

Microfluidic perfusion systems were employed to replicate dynamic physiological environments by regulating fluid flow, nutrient transport, and shear forces around cells. These systems were found to offer improved physiological relevance compared to static cultures, which lacked continuous medium exchange and often led to uneven nutrient distribution, abnormal cellular morphology, and reduced long-term viability [148,149,150,151,152,153,154,155,156]. However, maintaining stable perfusion conditions remained difficult, as flow consistency was affected by pump drift, changes in medium viscosity, channel obstruction, and cell-induced alterations in hydraulic resistance. Such variability frequently introduced experimental drift, which limited reproducibility in extended organ-on-chip studies [157,158,159,160,161,162,163].

To address these limitations, AI was increasingly incorporated into perfusion platforms, enabling adaptive flow control and automated interpretation of biological feedback. As illustrated in Figure 2, pump rates and valve settings were regulated using reinforcement learning and model predictive control frameworks, which responded in real time to input from flow, pressure, and oxygen sensors. By adjusting operational parameters based on system fluctuations, these algorithms maintained target shear stress and nutrient levels over long durations, with reduced need for manual correction, even during changes in cell density or medium composition. Machine-learning models were also applied to enhance analytical capabilities. Real-time image and biosensor data were processed using deep-learning algorithms to detect morphological changes, compromised barrier integrity, apoptotic activity, or altered migration behavior. Additional data collected from metabolic sensors and electrochemical probes were interpreted using supervised-learning techniques to infer shifts in cellular state [149,164,165,166]. When early deviations were detected, flow conditions were automatically modified to restore the intended environment, achieving a level of homeostasis that was difficult to maintain through manual adjustments [167,168,169,170,171,172].

AI further contributed to the design and optimization of perfusion architectures. Instead of relying on computationally intensive CFD simulations, surrogate models and neural-network approximations were used to predict flow distributions, nutrient gradients, and shear-stress concentrations within complex channel networks [173,174]. These tools were employed to design multi-branch vascular analogs, immune-cell migration platforms, and organ-specific microenvironments with greater precision. In systems containing multiple cell types, AI models were used to estimate how interactions among epithelial, endothelial, and immune cells influenced overall flow behavior, guiding the layout of microchannels that supported balanced culture conditions across all compartments [175,176,177,178]. For translational applications, AI-enhanced perfusion systems were used to improve the accuracy of drug-response assays by maintaining stable environmental conditions during drug exposure. Concentration profiles and the accumulation of drug metabolites were predicted using machine-learning models, allowing dynamic flow modulation to simulate in vivo pharmacokinetics. These real-time adjustments helped reduce variability and improve the predictive value of disease models used in preclinical research [179,180].

Despite these developments, certain limitations remained. Variability in biological data introduced uncertainty into training sets, while sensor noise and environmental shifts affected model accuracy. In many cases, AI models lacked mechanistic interpretability, which made it challenging to link learned outcomes with underlying biological processes. Future work is expected to focus on integrating physics-informed neural networks with experimental perfusion data to build models that combined mechanistic insight with data-driven prediction, improving reliability across diverse organ-on-chip systems.

### 3.4. Integration with Sensors and Actuators

Microscale sensors were embedded into biomedical devices to enable real-time awareness of the cellular environment. This was viewed as a crucial step in achieving responsive control in biological systems [181,182,183,184,185]. Traditional open-loop or static setups were unable to follow fast shifts in conditions like oxygen or nutrient levels, which made them less useful for experiments that needed continuous feedback. To collect more detailed data, sensors for chemical, thermal, electrical, and mechanical signals were integrated into microfluidic platforms. Figure 2D is added to depict the arrangement of sensors, actuators, and signal-transfer components within a typical microscale system. These sensors measured things like pH, oxygen concentration, glucose, temperature, and even electrophysiological activity. Although useful data were obtained, the speed and volume of measurements made it hard to interpret results manually. Errors and signal drift became more common over time [64,184,186,187,188,189].

To improve control, actuators were added to modify flow, pressure, heat, or local drug concentration in response to environmental changes. However, when sensor signals were linked to actuators through fixed rules, the system often failed to adapt, since biological behavior did not always follow predictable patterns. To solve this, AI was introduced. Sensor data were processed using machine-learning models that were trained to recognize changes in cell health, such as hypoxia, stress, or membrane breakdown [190,191,192,193,194]. These models passed predictions to control algorithms that adjusted actuators in real time. Flow, stimulation, or drug release was modified to bring conditions back to target levels. This created closed-loop systems that stayed stable even when cells or media changed over time.

Deep learning was also used to catch failures. Algorithms scanned incoming signals to detect odd patterns caused by blocked channels, worn actuators, or sensor failure. When something unusual was found, the system was able to correct itself or change settings to prevent bigger problems. This made long-term experiments more reliable and reduced the need for manual checks. AI was also helpful in designing systems. Instead of running lengthy simulations, neural networks were trained to predict how layout choices, such as where sensors and actuators are placed, would impact performance. In organ-on-chip models, this was used to tune mechanical stimulation for heart or muscle tissue. For wearable devices, AI has helped improve signal quality and power consumption during prolonged operation.

Even with these advances, some problems remained. Data from different sensors were not always formatted the same way. Actuator behavior may vary due to the manufacturing process. Furthermore, sometimes, it was unclear why the AI made certain choices. To move forward, researchers aimed to combine AI with biological models so that control strategies could be both data-driven and grounded in fundamental understanding.

## 4. Emerging Role of AI in Micro/Nanodevice Systems

AI is rapidly reshaping the landscape of micro- and nanodevice technologies, offering powerful new capabilities that extend well beyond traditional sensing and actuation. By leveraging advanced computational frameworks including ML, DL, and reinforcement learning (RL), researchers can now design micro/nano systems that are not only responsive but also adaptive, predictive, and increasingly autonomous [58,197]. These capabilities are critical for enhancing biomedical systems that must interpret complex biological data, make real-time decisions, and respond dynamically to changing physiological conditions [198]. AI enables micro/nanodevices to perform real-time control of critical parameters such as fluid flow, temperature, and chemical gradients, ensuring stable and reproducible microenvironments. In parallel, AI algorithms, particularly those based on deep neural networks are being deployed for high-throughput analysis of imaging data, biosensor signals, and cell behavior, allowing for rapid interpretation and classification that far surpasses manual methods in both speed and accuracy [83,188,199]. Also, AI facilitates intelligent system design through generative algorithms that can optimize microchannel geometries, predict material behaviors, and simulate device performance before fabrication. These approaches reduce prototyping cycles and enhance design robustness. Collectively, AI is transitioning micro/nanodevice platforms from passive diagnostic tools to active, self-optimizing systems poised to revolutionize drug discovery, precision medicine, and next-generation diagnostics [200,201].

### 4.1. AI for Real-Time Control

**Figure 3:**
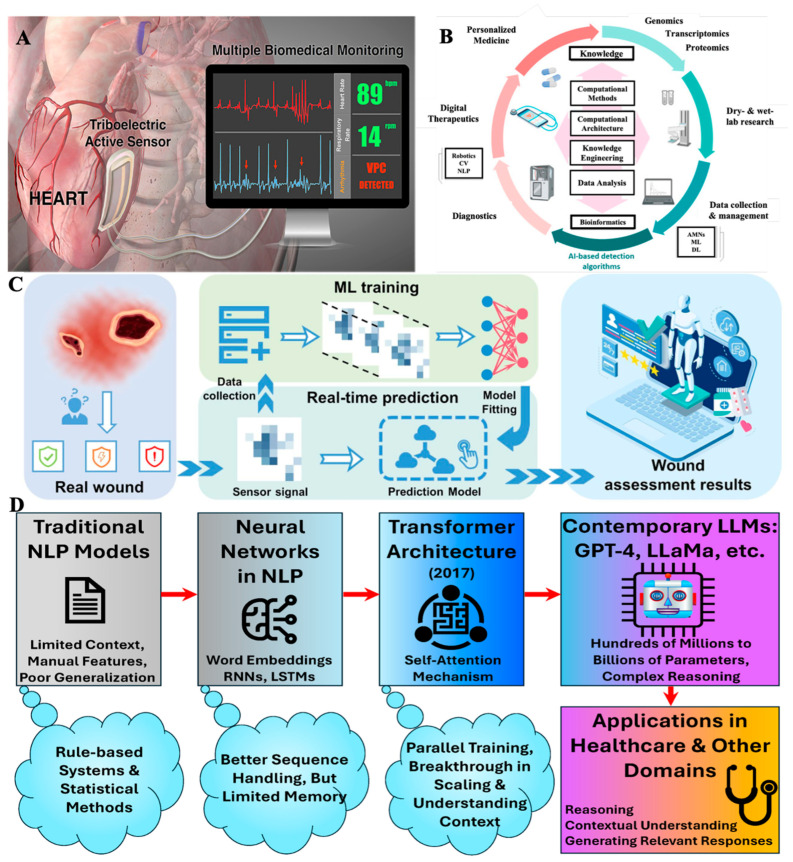
Emerging integration of AI and biomedical technologies for healthcare applications. (**A**) Schematic of a triboelectric active sensor for real-time biomedical signal monitoring and detection of cardiovascular events. The figure was reproduced with permission from [206], with a permission from ACS Publications, 2016, copyright license number (6118551468054). (**B**) Exploded-view illustration highlighting the structure and role of computational methods, bioinformatics, and AI-based algorithms in personalized medicine. The figure was reproduced with permission from [215], under a Creative Commons by Non-Commercial No Derivative works (CC-BY-NC ND 4.0) license, published by MDPI, 2022. (**C**) Workflow of intelligent wound management, showing the process from sensor signal acquisition and machine learning (ML) training to real-time prediction and personalized wound assessment. The figure was reproduced with permission from [219], with a permission from Elsevier, 2022, copyright license number (6118570218721). (**D**) Evolution of natural language processing (NLP) models: from traditional rule-based/statistical approaches to neural networks, the introduction of transformer architecture, and the rise in contemporary large language models (LLMs) such as GPT-4 and Llama, enabling advanced reasoning, contextual understanding, and applications in healthcare and other domains. The figure was reproduced with permission from [237], under a Creative Commons by Non-Commercial No Derivative works (CC-BY-NC ND 4.0) license, published by MDPI, 2025.

Integrating AI into micro- and nanoscale biomedical systems has been widely recognized as a critical advancement in the evolution of intelligent control architectures. Conventional strategies, such as rule-based logic or open-loop frameworks, have typically been limited by their inability to adapt to dynamic biological processes or environmental variability [202]. Such rigidity often results in suboptimal or unstable outcomes in systems requiring precise and sustained control over physiological parameters, such as organ-on-chip platforms, dynamic drug testing environments, and long-term tissue cultures [129,203]. To overcome these limitations, AI-driven control has been introduced to enable real-time adaptation, allowing the system to respond intelligently to changing conditions and complex feedback loops [58]. Figure 3A showed how an AI-assisted cardiac sensor system was used to monitor physiological signals during real-time control.

Within these platforms, advanced AI techniques, most notably reinforcement learning (RL), model predictive control (MPC), and adaptive fuzzy logic, have been implemented to manage multi-input, multi-output biological systems [204,205,206]. These algorithms have been trained or calibrated using historical system data or continuous real-time measurements, allowing predictive fluid flow, temperature, shear stress, and nutrient distribution adjustments. For example, RL agents have been utilized to fine-tune microfluidic pump speeds to maintain consistent shear forces across endothelial cell layers or regulate oxygen gradients within 3D tissue constructs [207,208]. Similarly, automated compensation has been performed to stabilize pH and osmolarity through real-time modulation of chemical delivery rates. By applying these learning-based strategies, control precision and system resilience have been substantially improved.

A defining feature of AI integration has been the inclusion of autonomous diagnostics and fault-tolerant behavior. System anomalies, such as sensor drift, channel occlusions, or biological instability, have been detected and corrected through built-in intelligence, reducing experimental error and improving reproducibility over long durations [209,210]. Furthermore, deploying edge computing and low-power AI hardware has enabled control logic to be embedded directly within portable, wearable, or implantable micro-devices. As a result, these platforms have functioned independently of external processing units, enabling truly closed loops and autonomous operation.

This convergence of AI algorithms with microdevice hardware has enabled the development of next-generation biomedical tools. Intelligent diagnostic platforms, precision therapeutic delivery systems, and self-regulating research models have been successfully demonstrated [211]. In summary, by replacing static control logic with adaptive, learning-based systems, AI has transformed micro/nanodevice platforms into interactive and autonomous entities capable of meeting personalized medicine demands, high-throughput experimentation, and real-time physiological interfacing.

### 4.2. AI for Data Analysis

With the rapid evolution of micro- and nanodevice platforms, increasingly large, complex, and multimodal datasets have been generated, encompassing high-resolution imaging, biosensor outputs, and dynamic time-series signals. The interpretation of such datasets has traditionally required labor-intensive, expert-driven analysis, often subject to bias and variability. AI, particularly ML techniques, has been integrated to automate and standardize data interpretation. Convolutional neural networks (CNNs), in particular, have been employed for biomedical image analysis, where tasks such as cell counting, morphological classification, and fluorescence quantification have been performed with high accuracy [199,212,213,214,215]. Subtle phenotypic shifts, such as cytoskeletal reorganization or nuclear shape changes, have been detected by these models, often surpassing the resolution and consistency of human observers [216,217]. As a result, more profound insight into early indicators of drug responses or disease progression has been achieved. Figure 3B illustrated how multimodal biomedical datasets were processed through AI-based computational pipelines for integrated interpretation.

In functional microdevices, including cardiomyocyte-on-chip platforms, contractile behavior has been analyzed from video recordings through AI-based methods [218,219]. Parameters such as beat frequency, contraction velocity, and arrhythmia profiles have been automatically extracted and evaluated. In parallel, unsupervised algorithms such as Gaussian Mixture Models (GMMs) and k-means clustering have been applied to categorize cellular responses, metabolic profiles, or behavioral signatures without predefined labels [220]. These approaches have allowed biomarker discovery, phenotypic subtyping, and treatment stratification to be conducted with reduced human oversight and improved analytical depth [221].

Beyond single-data-stream interpretation, AI has integrated multimodal datasets, including optical, mechanical, electrical, and chemical signals, within unified analytical frameworks. This approach has proven particularly effective in organ-on-chip and tumor-on-chip systems, where cellular behavior is influenced by a complex interplay of real-time factors [179,222]. Through such fusion, comprehensive mechanistic insight into cellular dynamics and intercellular interactions has been enabled.

The interpretability, accuracy, and reproducibility of micro/nanodevice data have been significantly improved through AI-driven analytics. Predictive modeling of drug effects, disease trajectories, and cellular responses has been enhanced, while integration with real-time feedback systems has laid the foundation for adaptive biomedical platforms [199,223]. As these intelligent frameworks become further embedded in experimental workflows, personalized diagnostics and autonomous systems are expected to be increasingly realized.

### 4.3. AI for Optimization and Design

The design, optimization, and fabrication of micro- and nanodevices have been increasingly transformed by adopting AI methodologies. The growing complexity of device architectures and the demand for application-specific performance have been recognized as primary incentives for this shift. Traditional workflows, often dependent on iterative manual adjustments, prototyping, and experimental validation, are labor-intensive and inefficient [87,208]. By contrast, AI-based design strategies have been enabled through historical datasets, numerical simulations, and prior experimental outcomes, allowing predictive, adaptive, and data-driven approaches to replace trial-and-error engineering. Figure 3C presented how sensor-derived wound signals were used in machine-learning workflows for prediction and optimization.

In optimizing device geometry, supervised learning models and algorithms such as genetic algorithms and Bayesian optimization have been applied to predict microchannel configurations for targeted functions, including fluid mixing, cell trapping, and gradient formation. Non-intuitive parameter combinations that yield enhanced performance have been identified, guiding engineers toward superior solutions [224,225,226]. Generative design frameworks, including variational autoencoders (VAEs) and generative adversarial networks (GANs), have been employed to produce device architectures that satisfy predefined functional outputs such as shear stress profiles, residence times, or spatial cell distributions [227]. As a result, the design space has been expanded far beyond the limits of conventional CAD-driven exploration. By enabling tailored control over flow patterns and mechanical forces, these geometry optimizations directly improve the fidelity of biological experiments within such devices.

Parallel advances have been achieved in materials discovery and optimization. ML models trained on chemical structures, mechanical properties, and processing parameters have been used to predict elasticity, hydrophilicity, degradation rates, and biocompatibility [228]. These predictions have enabled rapid screening of candidate materials and tailoring substrates and coatings to specific biomedical applications [229]. By reducing experimental screening requirements, discovery timelines have been shortened, and material choices have been more closely aligned with functional device requirements.

AI has also facilitated multi-objective optimization, allowing trade-offs among competing performance criteria to be quantitatively balanced [230]. Examples include reconciling the need to minimize shear stress for cell viability with the desire to maximize mixing efficiency for reagent delivery. With the continued expansion of open-source datasets, simulation libraries, and computational resources, the role of AI in intelligent biomedical device design is expected to become increasingly central [231,232,233]. Together, advances in AI-driven geometry, materials, and multi-objective optimization position AI not merely as a design aid but as the unifying foundation for creating next-generation biomedical micro/nanodevices, systems whose complexity can be optimized with unprecedented precision and adaptability.

### 4.4. Relevant AI Models and Outlook

The adoption of AI across micro- and nanodevice platforms has been driven by modern biomedical systems’ growing complexity and data intensity. These necessitate analytical and control strategies that surpass conventional rule-based methods [106]. A diverse range of AI models has been adapted according to these systems’ data type and functional requirements. For image-centric applications, CNNs have achieved high accuracy in segmentation, classification, and spatiotemporal tracking of biological features in microscopy and organ-on-chip imaging [234]. For non-image datasets, unsupervised learning methods such as GMMs and k-means clustering have been applied to detect patterns in biosensor outputs, single-cell measurements, and metabolomic profiles without requiring labeled datasets [235,236,237]. By combining these approaches, heterogeneous and high-dimensional outputs from micro/nanodevices have been transformed into integrated, interpretable datasets, enabling more accurate, comprehensive, and biologically relevant insights to be extracted in real time. Figure 3D summarized the progression of AI model architectures and showed how these frameworks were applied to micro/nanodevice systems.

Beyond data interpretation, AI has also enabled adaptive and autonomous control within micro/nanodevice systems. RL algorithms have been utilized to adjust microfluidic flow rates, thermal conditions, or drug delivery profiles in response to real-time feedback and reward signals [238,239]. Such capabilities have been considered essential for achieving closed-loop regulation in applications including tissue culture and personalized therapeutic monitoring. In parallel, classical machine learning models, including support vector machines (SVMs), decision trees, and random forests, have continued to be applied where structured datasets and interpretability are prioritized [240,241,242]. More recently, generative models such as variational autoencoders (VAEs) and generative adversarial networks (GANs) have been incorporated to generate synthetic datasets, optimize device configurations, and augment simulation environments [243,244]. By integrating these varied AI approaches, biomedical micro/nanodevice platforms have been enabled to operate with greater adaptability, efficiency, and precision, supporting real-time decision-making and the creation of innovative device designs that meet complex biological requirements.

Concurrent advances in hardware and software infrastructure have facilitated the practical realization of these AI capabilities. Edge AI processors, low-power embedded computing modules, and cloud-based training pipelines have been developed to enable on-device inference and continuous learning without reliance on external servers [245,246]. Through this convergence of algorithms and hardware, bio-interfacing devices have been enabled to autonomously sense, interpret, predict, and respond to physiological and environmental cues in real time. As these integrated systems have matured, they have been positioned to form the foundation for next-generation diagnostic platforms, adaptive drug screening systems, and personalized medicine technologies [247,248]. Collectively, these developments have established AI-enabled micro/nanodevices as a transformative class of biomedical tools, capable of redefining how biological data is processed and acted upon to achieve fully autonomous, precision-driven healthcare solutions.

### 4.5. AI-Driven In Silico Modeling and Discovery

In silico methods supported the development of micro- and nanodevice systems by enabling virtual testing, material screening, and molecular-level prediction before experimental fabrication. Machine-learning models were utilized to predict material properties, including elasticity, degradation rate, hydrophilicity, and biocompatibility, based on molecular descriptors and structural inputs. These approaches reduced the need for extensive laboratory screening and helped identify substrates or coatings suitable for micro- or nanorobotic operation [249,250,251]. AI techniques were also applied to molecular and biochemical interactions relevant to biosensing. Deep-learning models and graph-based neural networks were used to predict receptor–ligand binding, protein–surface interactions, and molecular docking outcomes. These predictions were used to guide the selection of recognition elements, surface chemistries, and binding motifs for integrated sensors. In parallel, bioinformatics frameworks were employed to analyze genomic, proteomic, and metabolomic datasets, identifying biomarkers suitable for detection by micro- or nanodevices [252,253,254,255]. Drug–target interactions, toxicity profiles, and therapeutic response signatures were predicted using supervised learning and generative models, enabling the tailoring of microdevice platforms for specific diagnostic or drug-screening applications [256,257,258]. Through these in silico tools, device development was accelerated and biological compatibility was improved by incorporating computational predictions prior to physical prototyping. These methods complemented AI-driven control, analysis, and design strategies described in earlier subsections, creating a unified computational ecosystem that supported the entire micro/nanodevice development pipeline.

## 5. Application in Biomedical and Life Science Research

Micro- and nanodevices were increasingly used in biomedical and life science research, as their ability to manipulate cellular and molecular environments with sub-micrometer precision was widely acknowledged. Their small size, biocompatibility, and compatibility with high-throughput systems were recognized as key enablers for applications ranging from drug screening to biosensing [188,259,260]. Spatial and temporal control of biological variables was combined with integrated sensing and actuation, positioning these devices as effective intermediaries between simplified in vitro models and more physiologically relevant in vivo systems. Their value extended beyond technical novelty; they were regarded as transformative tools capable of addressing long-standing limitations in conventional experimental methods [261,262,263]. Traditional 2D cultures often failed to capture complex biological dynamics, while animal models introduced translational limitations and ethical concerns due to interspecies variability. In contrast, device-based systems were designed to mimic physical forces, fluid shear, and multicellular interactions, enabling more accurate modeling of organ-level behavior and disease mechanisms [226,264,265]. Furthermore, their capacity to deliver analytical outputs in real time supported data-rich experimentation with potential to inform both fundamental biology and translational efforts.

However, several challenges remained. The complexity of device fabrication, absence of standardized protocols, and issues with scalability continued to limit broader clinical and industrial application. In parallel, the volume and complexity of data produced by these systems created a need for advanced computational approaches. This convergence between micro/nanotechnology and AI was increasingly recognized as essential for effective interpretation and control [266,267]. To address these demands, AI-based computational strategies were adopted. Machine-learning models were employed to analyze pharmacological responses, classify disease phenotypes, evaluate single-cell behavior, and process biosensor data in real time. These techniques allowed device outputs to be linked with adaptive analytics, improving the interpretation of biological signals. As a result, AI was embedded into downstream biomedical workflows, enhancing precision and responsiveness in both research and diagnostic contexts [268,269,270]. Despite technical and computational constraints, progress across multiple domains demonstrated strong interdependence. Developments in drug screening contributed to better disease modeling; insights from cell heterogeneity supported the expansion of single-cell analysis; and sensing technologies enabled biological signals to be converted into actionable feedback [271,272]. Rather than functioning as stand-alone innovations, micro/nanodevices were embedded within an interconnected and evolving technological ecosystem, one increasingly viewed as foundational to the future of biomedical science, diagnostics, and therapeutics.

### 5.1. Drug Screening

**Figure 4:**
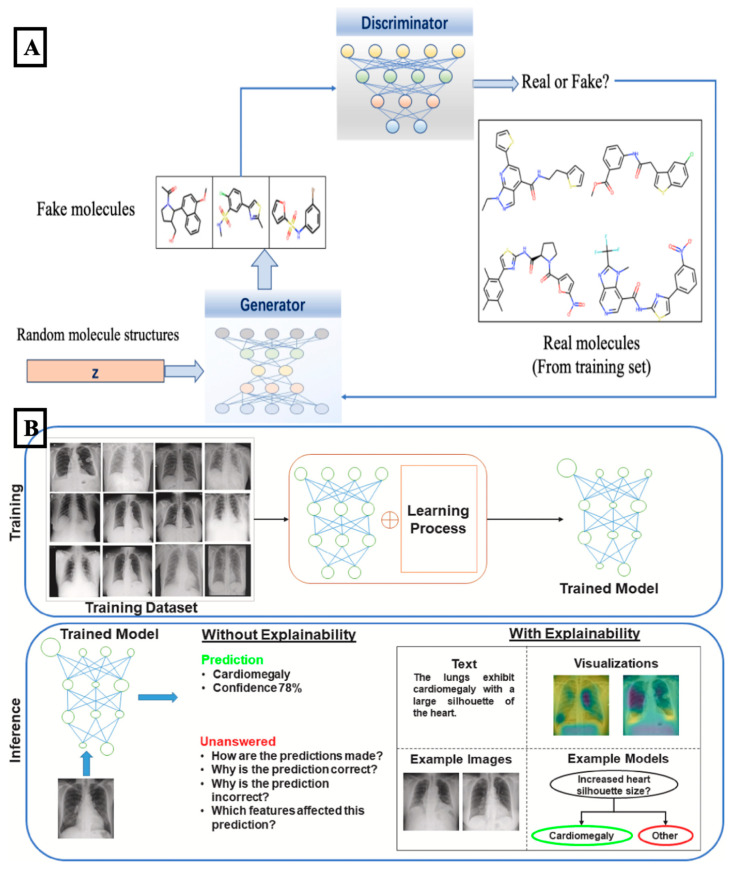
Applications of deep learning and explainable artificial intelligence (XAI) in biomedical research and sensing. (**A**) A schematic of drug-design workflows using generative adversarial networks (GANs) was shown. The generator created novel molecular structures from latent vectors, and the discriminator distinguished between fake and real molecules in the training dataset and provided feedback. The figure was reproduced with permission from [276], under a Creative Commons By–Non-Commercial–No Derivative Works (CC-BY-NC-ND 4.0) license, published by MDPI, 2022. (**B**) The role of XAI in medical image analysis was illustrated. A model was trained using chest X-ray datasets, and its predictions were explained by text-based and visualization-based methods. The explanations highlighted features such as cardiomegaly, which enabled users to interpret the model’s decision-making process. The figure was reproduced with permission from [281], under a Creative Commons By–Non-Commercial–No Derivative Works (CC-BY-NC-ND 4.0) license, published by Elsevier, 2023.

Conventional preclinical testing was inefficient because static cultures lacked physiological signs and animal models failed to capture human-specific responses, leading to high drug abrasion rates during drug development. Micro/nanodevices were applied to construct physiologically relevant microenvironments with real-time, low-volume, and high-throughput pharmacological assessment. Organ-on-chip systems were designed to reproduce organ-level mechanics, microvascular perfusion, and tissue–tissue interfaces [264]. Multi-organ chips were further developed to capture systemic pharmacokinetics and drug interactions, offering greater predictive power. AI-based methods were adopted to analyze the large datasets generated by these systems. Machine-learning models were used to interpret contractility signals, barrier-function measurements, fluorescence-based toxicity readouts, and image sequences of tissue response. Predictive algorithms estimated dose–response curves, toxicity trends, and therapeutic windows with improved consistency. Figure 4A showed how generative models were used to create and evaluate molecular structures, supporting automated discovery workflows relevant to drug-screening applications. Reinforcement-learning strategies were applied to adjust flow rates or drug-delivery schedules in real time to maintain stable physiological conditions during experiments. These approaches strengthened the reliability of drug-screening platforms and helped reduce user-dependent variability. However, limitations were also apparent. Standardization across platforms was lacking, reproducibility between laboratories was inconsistent, and regulatory pathways for chip-based arrangements remained uncertain [273,274,275,276,277]. In addition, while micro/nanodevices successfully mimicked localized tissue responses, long-term culture stability and incorporation of systemic immune interactions were not yet fully achieved. Despite these challenges, drug-screening platforms laid the groundwork for more complex disease models, where similar architectures were adapted to recreate pathological states rather than healthy physiology.

### 5.2. Disease Modeling

Micro- and nanodevices were increasingly applied to recreate disease-specific microenvironments, as animal models often failed to accurately represent human pathophysiology. Systems incorporating vascular-like networks, extracellular matrix analogs, and controlled chemical gradients were employed to simulate conditions associated with cancer progression, metabolic disorders, and neurological diseases. Patient-derived cells were used in glioblastoma-on-chip models to investigate tumor invasion, angiogenesis, and resistance to therapies. Similarly, islet-on-chip platforms and blood–brain barrier devices were designed to study diabetes and neurodegenerative conditions [278,279,280,281,282]. To support analysis, AI-based tools were introduced. Deep-learning models were trained to detect changes in cell shape, vascular remodeling, and local oxygen levels that were difficult to evaluate manually. Image-based networks were employed to identify tumor invasion fronts, therapy response patterns, and early signs of metabolic disruption. Figure 4B presented an example of medical-image analysis, where explainable AI was applied to improve interpretation of disease-related features within model systems. Time-series models were also used to process electrophysiological data and biochemical sensor outputs, allowing disease states and progression rates to be classified more accurately. These AI-assisted methods improved sensitivity and reduced human bias during interpretation. Despite these improvements, certain limitations remained. Immune and stromal cell types were often underrepresented. Variability between patients was difficult to fully capture, and larger-scale modeling efforts still required improvement in scalability [271,275,283,284,285]. Even so, the combination of advanced microdevice platforms with AI-driven analysis was shown to enhance the accuracy and reproducibility of disease modeling.

### 5.3. Single-Cell Analysis

**Figure 5:**
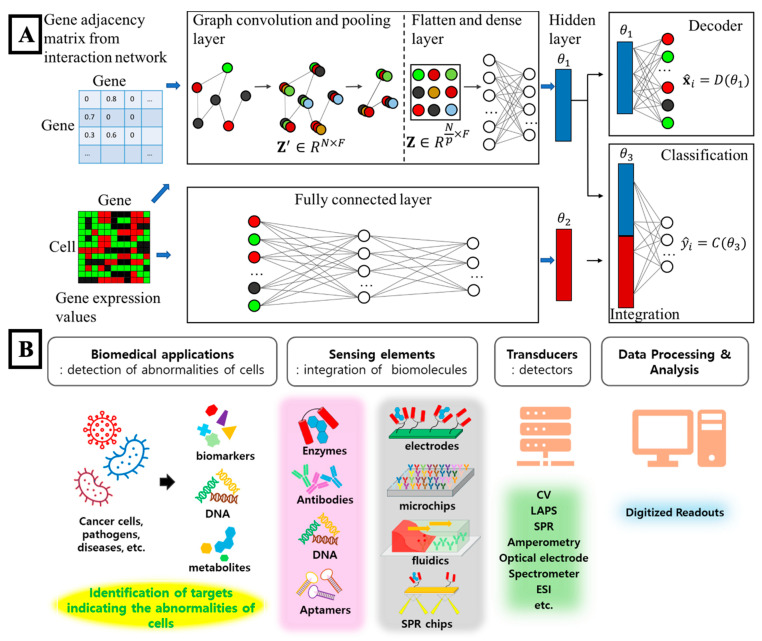
(**A**) The structure of a graph convolutional network (GCN) for single-cell classification was illustrated. Gene adjacency matrices and gene expression values were processed through graph-convolution, pooling, and dense layers. Hidden layers and decoder modules were used for feature extraction, and the extracted features were integrated for final classification. The figure was reproduced with permission from [292], under a Creative Commons By–Non-Commercial–No Derivative Works (CC-BY-NC-ND 4.0) license, published by Springer Nature, 2021. (**B**) Typical components of biosensor platforms were shown. Cellular targets interacted with biomolecules integrated into sensing elements, and these interactions were converted into signals by transducers such as electrodes, microchips, and fluidic interfaces. The signals were processed into digitized readouts for biomedical applications, including disease detection and biomarker identification. The figure was reproduced with permission from [298], under a Creative Commons By–Non-Commercial–No Derivative Works (CC-BY-NC-ND 4.0) license, published by MDPI, 2024.

Single-cell heterogeneity was recognized as a major driver of tissue-level function and disease behavior. Micro/nanodevices were used to isolate, capture, and manipulate individual cells, allowing rare phenotypes to be studied. Droplet-based systems such as Drop-seq enabled massively parallel transcriptomic profiling, while microwell arrays, nanostructured traps, and dielectrophoretic tweezers supported proteomic and functional assays [286,287,288]. AI-assisted computational techniques were adopted to manage the large datasets generated by these platforms. Clustering algorithms and dimensionality reduction models were used to classify rare cell populations, identify lineage trajectories, and detect functional subgroups within mixed samples. Figure 5A illustrated how graph-based learning was used to analyze gene interactions and single-cell expression patterns within complex datasets. Deep-learning–based image processing interpreted cell-membrane dynamics, calcium flux, and motility behavior with reduced manual intervention. These approaches improved the resolution of single-cell studies and enabled faster identification of biologically meaningful patterns. Challenges remained in linking molecular signatures to functional outcomes, integrating multimodal datasets, and improving reproducibility across analytical pipelines [289,290,291,292]. Nonetheless, AI-supported interpretation enhanced the utility of micro/nanodevices for assessing cellular diversity and mechanistic behavior at single-cell resolution.

### 5.4. Biosensing

Biosensing was recognized as a central capability in micro- and nanodevice applications, as it allowed for sensitive detection of chemical, biological, and physical signals. Micro- and nanostructured surfaces were engineered to harness plasmonic, electronic, or quantum effects, enabling label-free detection methods [293,294]. These sensing platforms were integrated with microfluidic systems to enable real-time and multiplexed assays, supporting both point-of-care diagnostics and continuous physiological monitoring. To interpret biosensor outputs effectively, AI-based signal-processing approaches were increasingly employed. Temporal signal patterns were classified, baseline drift was corrected, and noise was distinguished from meaningful features using machine-learning models. Deep-learning frameworks were used to identify viral markers, detect metabolic shifts, and interpret electrophysiological signals from wearable and implantable devices. Figure 5B demonstrated how sensing elements, transducers, and data-processing modules were combined to detect cellular abnormalities and biochemical markers in biomedical systems. Additionally, predictive algorithms were employed to estimate sensor degradation, correct systemic errors, and regulate closed-loop actuation within therapeutic systems. These tools improved overall detection accuracy and reduced the need for manual oversight during data interpretation. However, some limitations persisted. Long-term sensor stability, biofouling resistance, and clinical validation remained areas of concern [295,296,297,298]. Nevertheless, the integration of AI-supported analytics demonstrated how sensing, data interpretation, and responsive control could be combined within a single platform, laying the foundation for more autonomous biomedical systems [299].

Together, drug screening, disease modeling, single-cell analysis, and biosensing applications illustrated how micro/nanodevices redefined biomedical research by enabling physiologically relevant, ethically sustainable, and data-rich experimentation. Each domain reinforced the following: organ-level systems supported disease-specific modeling; recognition of heterogeneity directed focus to single-cell resolution; and advances in sensing closed the loop between biological detection and therapeutic action. Despite technical and translational challenges, convergence with computational and AI-driven approaches positioned micro/nanodevices as foundational technologies expected to evolve into fully autonomous, adaptive biomedical systems, ultimately shaping the next generation of diagnostics, therapeutics, and personalized medicine.

## 6. Challenges and Future Outlook

Micro/nanodevices were established as transformative tools in biomedical research, but their translation into routine clinical and industrial practice remained limited by persistent challenges. The features that granted these devices versatility, miniaturization, sensitivity, and functional integration, also introduced barriers to scalability, reproducibility, and regulatory acceptance [260,300]. Device fabrication was frequently limited by variability, while incorporating heterogeneous components such as biosensors, actuators, and living tissues created integration complexity. Moreover, the promise of AIas a driver of autonomous and adaptive device behavior was hindered by limitations in data availability, interpretability, and computational infrastructure [301,302,303].

These barriers were not merely technical but also extended into ethical, regulatory, and standardization domains, reflecting the multifaceted nature of translating experimental innovations into clinically deployable platforms. Nevertheless, the emergence of modular architectures, digital twins, interpretable AI, and edge computing illustrated that parallel solutions were already under development. Examining these challenges in detail and linking them to potential strategies envisioned a more precise trajectory toward the future generation of intelligent and autonomous micro/nanodevice systems [304,305,306].

### 6.1. Scalability, Reproducibility, and Integration Complex

Micro/nanodevice fabrication scalability was restricted because device performance was susceptible to manufacturing variability. While soft lithography, nanoimprint lithography, and 3D printing were widely employed for rapid prototyping, their translation to high-volume production remained problematic. Variations in channel geometry, surface roughness, and material consistency often introduced device-to-device differences, which resulted in significant biological variability in experimental outcomes. Integration complexity further compounded these limitations. Biomedical devices often require the co-fabrication of biosensors, actuators, electronics, and even living tissues, each with distinct material and operational demands [13,307,308,309]. Achieving seamless integration without interference proved difficult; for example, embedded electrodes altered fluid dynamics, while actuators occasionally disrupted electrical or thermal stability [187,310]. Emerging strategies such as modular fabrication architectures, self-aligned assembly, and standardized interface protocols were developed to overcome these issues. Digital twin simulations were increasingly employed to predict system-level performance before fabrication, thereby minimizing prototyping cycles and reducing costs [311,312,313]. Together with international standardization efforts, these approaches were expected to enhance reproducibility and accelerate industrial-scale adoption.

### 6.2. Barriers to AI Adoption in Micro/Nanodevice Systems

AI was identified as a transformative enabler for micro/nanodevice systems, providing capabilities in predictive analytics, adaptive control, and automated experimentation. However, several obstacles limited its adoption. The scarcity of large, high-quality annotated datasets restricted robust model development, since biological data collection was resource-intensive, heterogeneous, and prone to variability. The absence of standardized data formats and metadata further reduced reproducibility and cross-platform comparability [106,314,315]. Hardware constraints also limited AI deployment at the device level. Embedded processors were required to be compact, energy-efficient, and reliable, yet conventional AI hardware struggled to meet these demands while supporting advanced inference tasks. The interpretability of deep learning systems posed additional difficulties, as most operated as “black boxes,” which undermined regulatory approval and clinical trust. Moreover, risks of overfitting and model drift in dynamic biological environments necessitated frequent retraining, reducing long-term reliability [316,317,318,319]. Emerging solutions were being explored. Interpretable AI architectures, federated learning frameworks for privacy-preserving analysis, and the creation of open-access, annotated biomedical datasets were proposed to address data limitations. Advances in edge AI processors and neuromorphic computing began to mitigate hardware constraints, suggesting that embedded, autonomous decision-making within bio-integrated devices would soon become feasible.

### 6.3. Future Directions: Toward Intelligent and Autonomous Biological Platforms

The resolution of fabrication, integration, and AI-related challenges was anticipated to converge toward developing intelligent and autonomous micro/nanodevice platforms. Such systems were envisioned to operate as closed-loop environments, integrating high-resolution sensing, adaptive actuation, and AI-based decision-making to sustain physiologically relevant conditions over extended periods. In drug discovery, these platforms were expected to autonomously adjust dosing schedules or reagent delivery in response to real-time readouts of tissue responses [50,212,320,321]. In diagnostics, wearable and implantable systems were projected to continuously monitor biomarkers, predict disease onset, and initiate pre-programmed therapeutic interventions. However, implementing such intelligent systems also demanded parallel progress in ethical governance, regulatory approval, and cybersecurity protocols to ensure patient safety and societal trust. If these challenges were effectively addressed, the convergence of AI, innovative materials, and advanced micro/nanofabrication would redefine biomedical engineering [2,322]. In this way, micro/nanodevices were expected to transition from passive experimental tools into self-regulating, precision-driven healthcare platforms capable of operating with minimal human oversight. In summary, while scalability, integration, and AI adoption challenges persisted, parallel progress in fabrication strategies, computational frameworks, and standardization efforts demonstrated that solutions were already emerging. Each barrier was increasingly addressed through interdisciplinary advances, setting the stage for the next generation of intelligent biomedical platforms. This trajectory suggested that micro/nanodevices would evolve beyond experimental applications into autonomous, adaptive, and clinically relevant systems, ultimately shaping the future of diagnostics, therapeutics, and personalized medicine.

## 7. Conclusions

The convergence of AI with micro/nanodevice technologies has opened unprecedented boundaries in biomedical sensing, diagnostics, and therapeutics. Once limited to static tools, these platforms have evolved into dynamic systems capable of high-resolution monitoring, adaptive control, and autonomous decision-making. Advances in fabrication strategies, including photolithography, nanoimprinting, 3D printing, and self-assembly, have expanded the design landscape, enabling micro/nanorobots with architectures optimized for seamless biological integration. When combined with biocompatible materials, extracellular matrix-mimetic environments, and embedded sensors or actuators, these devices now achieve levels of interaction with living systems that were previously unattainable. AI has amplified this transformation by providing robust data interpretation, optimization, and closed-loop control frameworks. Machine learning and deep learning algorithms now support predictive modeling of biological processes, automated analysis of high-dimensional datasets, and intelligent regulation of dynamic microenvironments. These capabilities elevate micro/nanorobots beyond responsive platforms, positioning them as adaptive systems capable of learning from and continuously responding to complex biological contexts. However, despite remarkable progress, barriers remain. Scalability, reproducibility, data standardization, and regulatory acceptance continue to challenge clinical translation, while ethical, computational, and infrastructural constraints underscore the multifaceted nature of real-world deployment. Progress in modular fabrication, interpretable AI, edge computing, and standardized protocols indicates that viable solutions are already emerging. Looking ahead, the integration of AI-enabled micro/nanodevices into biomedical workflows promises to redefine personalized medicine, point-of-care diagnostics, and therapeutic interventions. By bridging the gap between in vitro modeling and in vivo functionality, these systems are poised to function as autonomous, precision-driven healthcare platforms. If the current barriers are systematically addressed, AI-integrated micro/nanorobots will advance from proof-of-concept demonstrations to foundational technologies, ushering in a new era of diagnostics, drug discovery, and adaptive therapeutics, ultimately transforming the landscape of biomedical engineering and clinical practice.

## Data Availability

There is no additional data for this work.
